# Essential Oils from *Monarda fistulosa*: Chemical Composition and Activation of Transient Receptor Potential A1 (TRPA1) Channels

**DOI:** 10.3390/molecules25214873

**Published:** 2020-10-22

**Authors:** Monica Ghosh, Igor A. Schepetkin, Gulmira Özek, Temel Özek, Andrei I. Khlebnikov, Derek S. Damron, Mark T. Quinn

**Affiliations:** 1 Department of Biological Sciences, School of Biological Sciences, Kent State University, Kent, OH 44242, USA; mghosh@kent.edu (M.G.); ddamron@kent.edu (D.S.D.); 2 Department of Microbiology and Immunology, Montana State University, Bozeman, MT 59717, USA; schepetkin@yahoo.com; 3 Department of Pharmacognosy, Faculty of Pharmacy, Anadolu University, 26470 Eskisehir, Turkey; gulmiraozek@gmail.com (G.Ö.); temelozek@gmail.com (T.Ö.); 4 Medicinal Plant, Drug and Scientific Research and Application Center (AUBIBAM), Anadolu University, 26470 Eskişehir, Turkey; 5 National Research Tomsk Polytechnic University, Tomsk 643050, Russia; aikhl@chem.org.ru; 6 Faculty of Chemistry, Tomsk State University, 634050 Tomsk, Russia

**Keywords:** *Monarda fistulosa*, essential oils, calcium flux, TRPA1, monoterpene, carvacrol, thymol, β-myrcene

## Abstract

Little is known about the pharmacological activity of *Monarda fistulosa* L. essential oils. To address this issue, we isolated essential oils from the flowers and leaves of *M. fistulosa* and analyzed their chemical composition. We also analyzed the pharmacological effects of *M. fistulosa* essential oils on transient receptor potential (TRP) channel activity, as these channels are known targets of various essential oil constituents. Flower (MEO_Fl_) and leaf (MEO_Lv_) essential oils were comprised mainly of monoterpenes (43.1% and 21.1%) and oxygenated monoterpenes (54.8% and 77.7%), respectively, with a high abundance of monoterpene hydrocarbons, including *p*-cymene, γ-terpinene, α-terpinene, and α-thujene. Major oxygenated monoterpenes of MEO_Fl_ and MEO_Lv_ included carvacrol and thymol. Both MEO_Fl_ and MEO_Lv_ stimulated a transient increase in intracellular free Ca^2+^ concentration ([Ca^2+^]_i_) in TRPA1 but not in TRPV1 or TRPV4-transfected cells, with MEO_Lv_ being much more effective than MEO_Fl_. Furthermore, the pure monoterpenes carvacrol, thymol, and β-myrcene activated TRPA1 but not the TRPV1 or TRPV4 channels, suggesting that these compounds represented the TRPA1-activating components of *M. fistulosa* essential oils. The transient increase in [Ca^2+^]_i_ induced by MEO_Fl_/MEO_Lv_, carvacrol, β-myrcene, and thymol in TRPA1-transfected cells was blocked by a selective TRPA1 antagonist, HC-030031. Although carvacrol and thymol have been reported previously to activate the TRPA1 channels, this is the first report to show that β-myrcene is also a TRPA1 channel agonist. Finally, molecular modeling studies showed a substantial similarity between the docking poses of carvacrol, thymol, and β-myrcene in the binding site of human TRPA1. Thus, our results provide a cellular and molecular basis to explain at least part of the therapeutic properties of these essential oils, laying the foundation for prospective pharmacological studies involving TRP ion channels.

## 1. Introduction

The genus *Monarda* L. (Lamiaceae) includes 20 species native to the United States [1] and is divided into two distinct subgenera, Monarda and Cheilyctis [2]*. M. fistulosa* (wild bergamot) has been used medicinally by Native American groups for treating colds, stomach pain, headache, wounds, and skin problems, among many other disorders [1]. In addition, extracts of *Monarda* L. spp. have been reported to exhibit antihelmintic, carminative, expectorant, febrifuge, rubefacient, and antimicrobial activities [3].

A number of phytochemical studies on *Monarda* species resulted in the identification of a wide spectrum of phytoconstituents, including phenolics [4,5], triterpenoids [6], monoterpene glycosides [7], carbohydrate, carotenoids, and vitamin C [8], with the phenolic complex containing flavonoids [5], anthocyanins, and phenolic acids. One compound specific to the *Monarda* genus is monardaein [9]. However, the phytochemical data reported in the literature are insufficient to individuate any chemotaxonomic marker [3]. Essential oils of *Monarda* species have been reported to be rich in oxygenated monoterpenes, leading to differentiation into the following chemotypes: thymol, carvacrol, borneol, and geraniol [10]. A literature survey regarding biological activities of the *Monarda* species essential oils and their major compounds, based on references [11,12,13,14,15,16,17,18,19,20,21,22,23,24,25,26,27,28,29], is summarized in Table 1.

Members of the calcium-permeable transient receptor potential (TRP) ion channel family respond to a wide variety of odorant phytochemicals. For example, TRPA1 is activated by mustard oil or allyl isothiocyanate (AITC) from the mustard plant [30], TRPM8 is activated by menthol from mint [31,32], and TRPV1 is activated by capsaicin from chili peppers [33]. Essential oil constituents, primarily monoterpenes, have been reported to exert beneficial physiological effects. For example, TRP channels of the vanilloid (TRPV) and melastatin (TRPM) subfamily have been reported to enhance the re-epithelialization of cutaneous wounds in the presence of β-caryophyllene [34]. Essential oils isolated from the genus *Hyptis* have been shown to exert antinociceptive effects [35], and rue phytochemicals have been shown to impact gustation [36] via potential interaction with TRPA1, TRPV1, and TRPM8. Finally, carvacrol, a monoterpene found in oregano, has been shown to be a potent vasodilator through activation of TRPV3 channels in rat smooth muscle cells [37].

TRPA1, the single ion channel in the ankyrin subfamily, is emerging as a significant target for several phytochemicals and related compounds with novel analgesic and anti-inflammatory properties [38]. Based on the reported anti-inflammatory properties of *M. fistulosa* extracts and the likely high monoterpene composition of essential oils from this plant, we hypothesized that TRPA1 might be one of its potential therapeutic targets because monoterpenes are known to target TRPA1. Thus, we evaluated these essential oils and their major constituents, including carvacrol, thymol, β-myrcene, α-terpinene, γ-terpinene, and *p*-cymene, for their ability to activate intracellular Ca^2+^ flux in HEK293 cells expressing TRPV1, TRPV4, or TRPA1. We found that *Monarda* essential oils activated TRPA1 ion channels but had no effect on TRPV1 or TRPV4 channels. Furthermore, TRPA1 agonist effects were primarily attributed to the monoterpene constituents (carvacrol, thymol, and β-myrcene).

## 2. Results

### 2.1. Essential Oil Composition

The chemical composition and biological activity of essential oils can be affected by many factors, including which part of the plant is used for essential oil isolation [39]. Indeed, we observed differences in the essential oil composition in flowers and leaves from *Ferula iliensis* [40] and *Hypericum perforatum* [41]. Thus, we extracted essential oils from *M. fistulosa* flowers (MEO_Fl_) and leaves (MEO_Lv_). The MEO_Fl_ and MEO_Lv_ extraction yields (v/w) were 1.3% and 4.4%, respectively, which are much higher than those reported previously by Mattarelli et al. (0.33–0.50%) [10]. The chemical composition of these essential oils was evaluated using gas chromatography-flame ionization detector (GC-FID) and gas chromatography-mass spectrometry (GC-MS) simultaneously, and Table 2 and Table 3 summarize the compounds identified, percentage composition, and relative retention indices (RRI) (compounds are listed in order of their elution).

A total of 45 distinct compounds was identified in *M. fistulosa* essential oils. Thirty-six and 34 compounds were identified in MEO_Fl_ and MEO_Lv_, respectively, representing >99.9% of the total essential oil composition. MEO_Fl_ and MEO_Lv_ were comprised mainly of monoterpenes (43.1% and 21.1%) and oxygenated monoterpenes (54.8% and 77.7%), respectively, with a high abundance of monoterpene hydrocarbons, including *p*-cymene (25.6% and 13.1%), γ-terpinene (6.8% and 2.5%), α-terpinene (3.8% and 1.7%), and α-thujene (2.4% and 1.3%), respectively. Major oxygenated monoterpenes of MEO_Fl_ and MEO_Lv_ included carvacrol (47.5% and 71.5%) and thymol (3.1% and 3.3%), respectively. Thirty-nine other compounds were present at concentrations <1.6%.

Comparison of the chemical profiles of MEO_Fl_ and MEO_Lv_ with *Monarda* essential oils reported previously showed that they all contained the common oxygenated monoterpene carvacrol. However, essential oils isolated from plants harvested from field rows contained relatively high amounts of *p*-cymene (35%) and 1-octen-3-ol (10%) and a low content of thymol (0.2%) [16].

Previously studied species of *Monarda* had essential oils almost exclusively comprised of oxygenated monoterpenes, with thymol and carvacrol as the main constituents (Table 1) [8,11]. *M. didyma* grown in Canada had thymol, γ-terpinene, carvacrol, and β-myrcene as the main constituents, while Canadian *M. fistulosa* essential oil contained geraniol, geranial, geranyl formate, and *p*-cymene [13]. In contrast, *M. fistulosa* cultivated in Italy was characterized by a high content of thymol (28–33%). Essential oils of *M. fistulosa* from the Krasnodar region of Russia contained *p*-cymene (32.5%), carvacrol (24.0%), thymol (12.6%), and the methyl ether of carvacrol (5.5%) [42]. The cultivation of *Monarda* species (*fistulosa, didyma, media,* and *menthifolia*) with a high content of thymoquinone, which has anticancer, anti-inflammatory, antidiabetic, and antioxidant activities, has also been reported [43,44,45,46], including methods for extraction of thymoquinone from *Monarda* [17,47].

### 2.2. Effect of M. fistulosa Essential Oils on TRP Ion Channels

**Figure 1:**
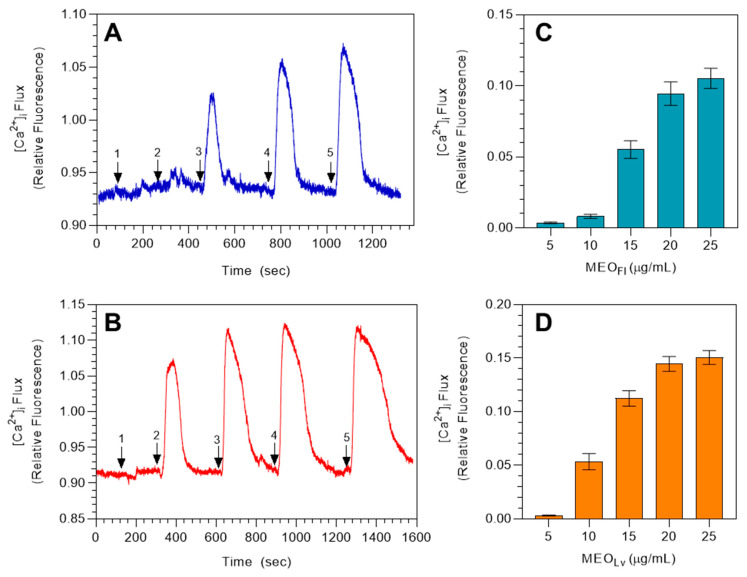
*Monarda* essential oils increase [Ca^2+^]_i_ in HEK transient receptor potential TRPA1 cells. Original representative traces demonstrating the dose-dependent effects of flower essential oil (MEO_Fl_) (**A**) and leaf essential oil (MEO_Lv_) (**B**). Arrows indicate the effects of 5 (#1), 10 (#2), 15 (#3), 20 (#4), and 25 (#5) µg/mL of the oils on [Ca^2+^]_i_ in HEK rTRPA1 cells. Summarized data for the dose-dependent increase in [Ca^2+^]_i_ are shown in (**C**) and (**D**). The data are represented as mean ± SEM (*n* = 90 cells) from 3 independent experiments.

Essential oils and their components have been reported previously to modulate TRP channel activity [48,49,50,51,52,53,54,55]. To investigate whether TRPA1 channels could be activated by *M. fistulosa* essential oils, we performed studies assessing changes in [Ca^2+^]_i_ in HEK 293 cells overexpressing TRPA1. HEK rTRPA1 cells were treated with increasing concentrations of MEO_Fl_ and MEO_Lv_, with Hanks’ balanced-salt solution (HBSS) washes included between each dose, as indicated in Figure 1A,B. Real-time calcium measurements revealed that *M. fistulosa* essential oils dose-dependently induced transient [Ca^2+^]_i_ influx. Transient changes between baseline to peak [Ca^2+^]_i_ induced by 20 and 25 µg/mL of MEO_Fl_ (0.095 ± 0.008 and 0.105 ± 0.007) and MEO_Lv_ (0.145 ± 0.007 and 0.150 ± 0.006) had similar magnitudes; hence, we chose 20 µg/mL as the working concentration for further experiments, since it was the concentration where the agonist effect plateaued. Analysis of the AITC (TRPA1 agonist)-induced increase in [Ca^2+^]_i_ confirmed the functional expression of TRPA1 channels in these cells. Summarized data showing the quantified transient increases in [Ca^2+^]_i_ induced by increasing concentrations of the essential oils are shown in Figure 1C,D for MEO_Fl_ and MEO_Lv_, respectively.

**Figure 2:**
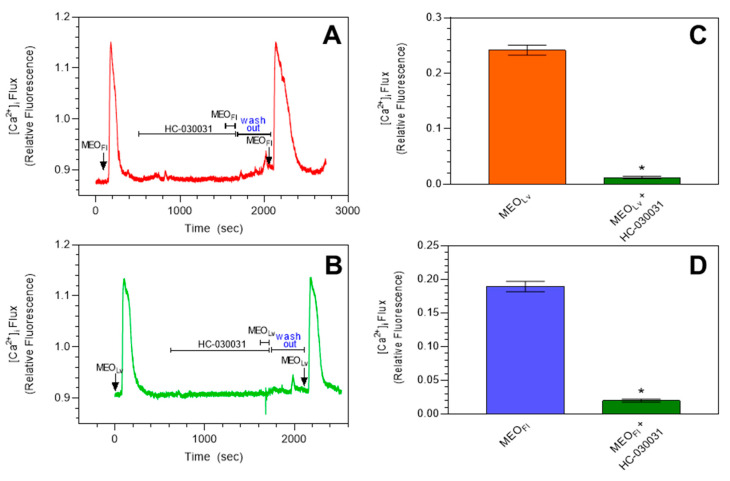
*Monarda* essential oil-induced increases in [Ca^2+^]_i_ are blocked by HC-030031 in HEK rTRPA1 cells. (**A**) and (**B**) are representative traces showing the effects of pretreatment with the selective TRPA1 blocker HC-030031 (20 µM) on the transient increase in [Ca^2^^+^]_i_ in HEK-rTRPA1 cells. Summarized data are shown in (**C**) and (**D**), respectively. The data are expressed as changes in the [Ca^2+^]_i_ influx from baseline to peak [Ca^2+^]_i_ and plotted as the mean ± SEM. * *p* < 0.0001 compared to [Ca^2+^]_i_ elicited after treatment with essential oils. Statistical analysis was performed using an unpaired *t*-test; *n* = 90 cells from 3 independent experiments.

To investigate whether *Monarda* essential oils elicited a transient increase in [Ca^2+^]_i_ exclusively through TRPA1 channels, we evaluated the specific TRPA1 antagonist HC-030031 [56] on the response to Monarda essential oils and found that the transient increases in [Ca^2+^]_i_ elicited via MEO_Fl_ and MEO_Lv_ were eliminated in HEK rTRPA1 cells when cells were pretreated with HC-030031 (20 µM) for 20 min, and reversible inhibition was confirmed by a subsequent transient increase in [Ca^2+^]_i_ elicited by the essential oils following washout of HC-030031 from the perfusion chamber with the control buffer (Figure 2). The *Monarda* essential oil-elicited transient increases in [Ca^2+^]_i_ pre- and post-HC-030031 treatments were of similar magnitude, further confirming the reversible antagonistic nature of HC-030031 inhibition.

**Figure 3:**
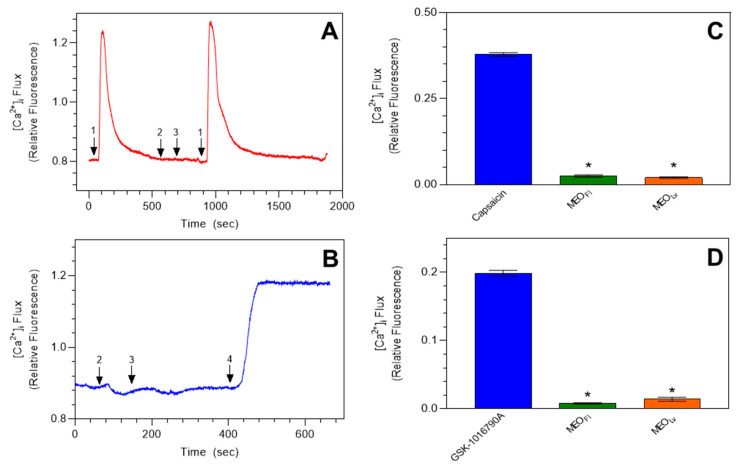
*Monarda* essential oil-induced increases in [Ca^2+^]_i_ are independent of TRPV1 and TRPV4 channels. Representative traces demonstrating the unaltered [Ca^2+^]_i_ baseline in HEK hTRPV1 (**A**) and HEK hTRPV4 (**B**) in the presence of MEO_Fl_ (arrow #2) and MEO_Lv_ (#3) (25 µg/mL). The TRPV1 agonist capsaicin (100 nM, #1) and the TRPV4 agonist GSK-1016790A (10 µM, #4) were used to confirm the expression of functional TRPV1 and TRPV4 channels in HEK cells. Summarized data are shown in (**C**) and (**D**), respectively. The data are expressed as the change in [Ca^2+^]_i_ influx from baseline to peak [Ca^2+^]_i_ and plotted as the mean ± SEM. * *p* < 0.0001 compared to the agonist-induced [Ca^2+^]_i_ influx. Statistical analysis was performed using one-way analysis of variance; *n* = 90 cells from 3 independent experiments.

To investigate whether *Monarda* essential oils activated members of the vanilloid group of TRP channels (V1 and V4), we performed real-time calcium imaging in HEK 293 cells stably overexpressing either hTRPV1 or hTRPV4 channels. Representative traces show that 20 µg/mL of MEO_Fl_ and MEO_Lv_ failed to induce any increase in [Ca^2+^]_i_ in HEK hTRPV1 and HEK hTRPV4 cells (Figure 3).

Capsaicin (TRPV1 agonist) and GSK-1016790A (TRPV4 agonist) were evaluated to confirm the functional expressions of TRPV1 and TRPV4, respectively [57,58]. The summarized data show the difference between the control agonist-induced [Ca^2+^]_i_ responses and those of the essential oils, which were inactive in HEK hTRPV1- and HEK hTRPV4-transfected cells (Figure 3).

**Figure 4:**
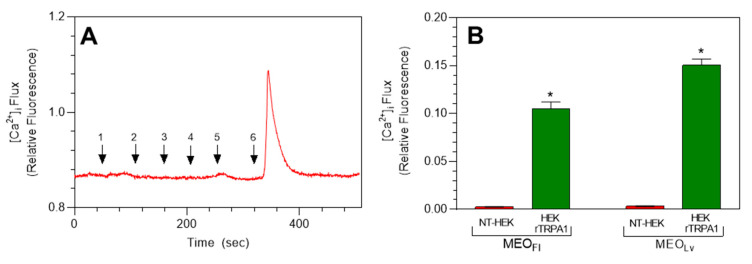
TRP agonist- and *Monarda* essential oil-induced increases in [Ca^2+^]_i_ are absent in parental HEK cells. Representative trace (**A**) showing the negligible effect in baseline [Ca^2+^]_i_ in parental HEK 293 cells treated consecutively with 100 µM of allyl isothiocyanate (AITC) (#1), 100 nM of capsaicin (#2), 10 µM of GSK1016790A (#3), 20 µg/mL of MEO_Fl_ (#5), and 20 µg/mL of MEO_Lv_ (#6). Receptor-effector coupling was confirmed by treating the cells with 25 µM of ATP (#6). Summarized data comparing the effects of MEO_Fl_ and MEO_Lv_ on [Ca^2+^]_i_ in parental HEK 293 and HEK rTRPA1 cells (**B**). The data are expressed as change in the [Ca^2+^]_i_ influx from baseline to peak [Ca^2+^]_i_ and plotted as the mean ± SEM. * *p* < 0.0001 compared to agonist-induced [Ca^2+^]_i_ influx. Statistical analysis was performed using two-way analysis of variance; *n* = 90 cells from 3 independent experiments.

To further confirm the selective agonist effects of *M. fistulosa* essential oils on TRPA1 channels, parental HEK 293 cells were subjected to real-time calcium flux analysis in the presence of MEO_Fl_ and MEO_Lv_, and representative traces illustrating the effects of TRP channel agonists and *M. fistulosa* essential oils on [Ca^2+^]_i_ in parental HEK 293 cells are shown in Figure 4. TRP channel agonists AITC (100 µM, TRPA1), capsaicin (100 nM, TRPV1), and GSK-1016790A (10 µM, TRPV4) showed no significant changes in baseline [Ca^2+^]_i_ in parental HEK 293 cells. Similarly, no changes in [Ca^2+^]_i_ were observed when parental HEK 293 cells were treated with either MEO_Fl_ or MEO_Lv_ (Figure 4A). Receptor-effector coupling and cell viability were confirmed using ATP (25 µM) to induce transient increases in [Ca^2+^]_i_ via purinergic receptor activation. Overall, the summarized data clearly demonstrate the significant differences in MEO_Fl_- and MEO_Lv_-induced changes in [Ca^2+^]_i_ in rTRPA1-transfected and parental HEK 293 cells (Figure 4B).

Although we did not evaluate the cytotoxicity of the *Monarda* essential oils, the ability of these extracts to activate TRPA1-dependent calcium flux at the indicated concentrations suggests that they were not toxic to the transfected cell lines, and no effects on cell viability were observed in our microscopy-based assays. Furthermore, the essential oils, as well as their constituent monoterpenes (see below), had no effect on the nontransfected/parental HEK 293 cell viability. Indeed, receptor-effector coupling of these cells after essential oil treatment was robust upon treatment with ATP, which induced a pronounced increase in [Ca^2+^]_i_ and demonstrated that the cells were still viable and functional after essential oil or monoterpene treatment.

### 2.3. Effects of Monarda Essential Oil Constituents on TRPA1 Activation

**Figure 5:**
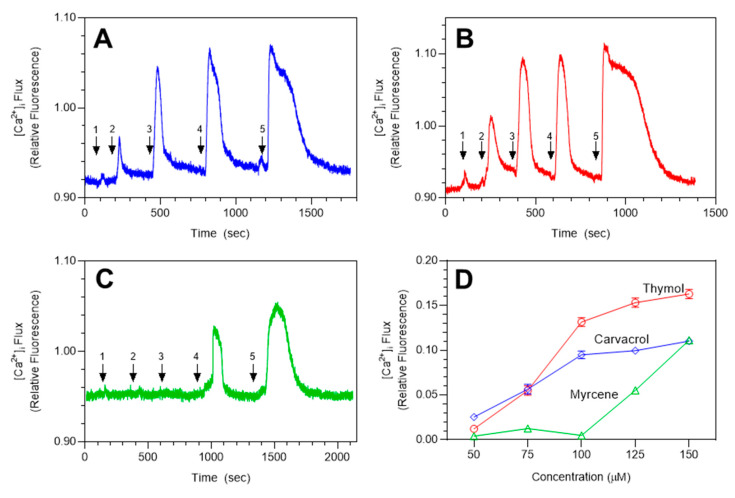
*Monarda* constituents carvacrol, thymol, and myrcene induce [Ca^2+^]_i_ increases in HEK rTRPA1 cells. Representative traces demonstrating the dose-dependent effects of carvacrol (**A**), thymol (**B**), and β-myrcene (**C**) (arrows #1–5 representing 50, 75, 100, 125, and 150 µM of each compound) on [Ca^2+^]_i_ in HEK rTRPA1 cells. Summarized data for the dose-dependent increases in [Ca^2+^]_i_ are shown in (**D**). The data are presented as the mean ± SEM (*n* = 90 cells) from 3 independent experiments.

Since *Monarda* essential oils activated TRPA1 channels, we next investigated the TRPA1-activating effects of their constituent monoterpenes to identify the active compounds. Sixteen commercially available compounds, including six major compounds (carvacrol, thymol, β-myrcene, α-terpinene, γ-terpinene, and *p*-cymene), were evaluated for agonist effects on [Ca^2+^]_i_ in HEK rTRPA1 cells; however, only carvacrol, thymol, and β-myrcene exhibited significant agonist activity (Table 4). As shown in Figure 5, increasing concentrations of carvacrol (Figure 5A), thymol (Figure 5B), and β-myrcene (Figure 5C) dose-dependently induced transient increases in [Ca^2+^]_i_ via the activation of TRPA1 channels. Thus, these three compounds, representing 50.2% of MEO_Fl_ and 75.6% of MEO_Lv_, had similar agonist activity as the whole essential oils themselves. Summarized line graphs showing the changes in [Ca^2+^]_i_ induced by these monoterpenes in HEK rTRPA1 cells are presented in Figure 5D. In contrast, α-terpinene, γ-terpinene, *p*-cymene, and ten minor constituents of the *Monarda* essential oils did not elicit significant changes in [Ca^2+^]_i_ in HEK rTRPA1 cells (Table 4).

**Figure 6:**
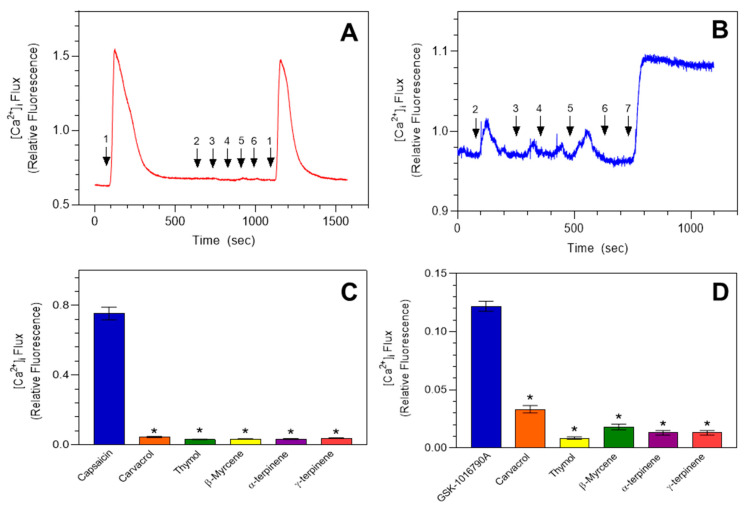
Monoterpenes do not induce increases in [Ca^2+^]_i_ in HEK cells expressing TRPV1 and TRPV4 channels. Representative traces demonstrating the unaltered [Ca^2+^]_i_ baseline in HEK hTRPV1 (**A**) and HEK hTRPV4 (**B**) in the presence of 100 µM of carvacrol (#2), thymol (#3), β-myrcene (#4), α-terpinene (#5), and γ-terpinene (#6). Capsaicin (#1), the MTRPV1 agonist, and GSK-1016790A (#7), the TRPV4 agonist, were used to confirm the expressions of the TRPV1 and TRPV4 channels, respectively. Summarized data are shown in (**C**) and (**D**) for TRPV1 and TRPV4 HEK cells, respectively. The data are expressed as change in the [Ca^2+^]_i_ influx from baseline to peak [Ca^2+^]_i_ and plotted as mean ± SEM. * *p* < 0.0001 compared to agonist-induced [Ca^2+^]_i_ influx. Statistical analysis was performed using one-way analysis of variance; *n* = 90 cells from 3 independent experiments.

Although MEO_Fl_ and MEO_Lv_ did not activate the hTRPV1 or hTRPV4 channels, we still evaluated the major monoterpene constituents of these essential oils in HEK hTRPV1 and hTRPV4 cells. Real-time [Ca^2+^]_i_ assessment in HEK hTRPV1 (Figure 6A) and hTRPV4 cells (Figure 6B) showed that 100 µM of carvacrol, thymol, β-myrcene, α-terpinene, and γ-terpinene had no effect on baseline [Ca^2+^]_i_ in either transfected cell line. We used 100 µM concentrations to evaluate the responses of these TRP channels, since this concentration fully activated TRPA1 and was clearly in the plateau of the dose response. As controls, capsaicin and GSK-1016790A were used to confirm the functional expression of TRPV1 and TRPV4, respectively. Summarized data showing the significant differences in [Ca^2+^]_i_ between the agonists and monoterpenes are shown in Figure 6C,D for HEK hTRPV1 and hTRPV4, respectively.

As with the experiments above using whole essential oil extracts, it is unlikely that the monoterpenes evaluated were cytotoxic, as no cytotoxicity was observed microscopically, and the cells responded robustly to the positive control agonists in all three transfected cell lines. In addition, we previously demonstrated that similar monoterpenes tested at 100 µM had no effect on human MonoMac-6 monocytic cell viability [59]. Normal AITC responses for HEK rTRPA1 cells were also observed after every experiment to confirm TRPA1 sensitivity and receptor-effector coupling (not shown in the figures).

### 2.4. Molecular Modeling

**Figure 7:**
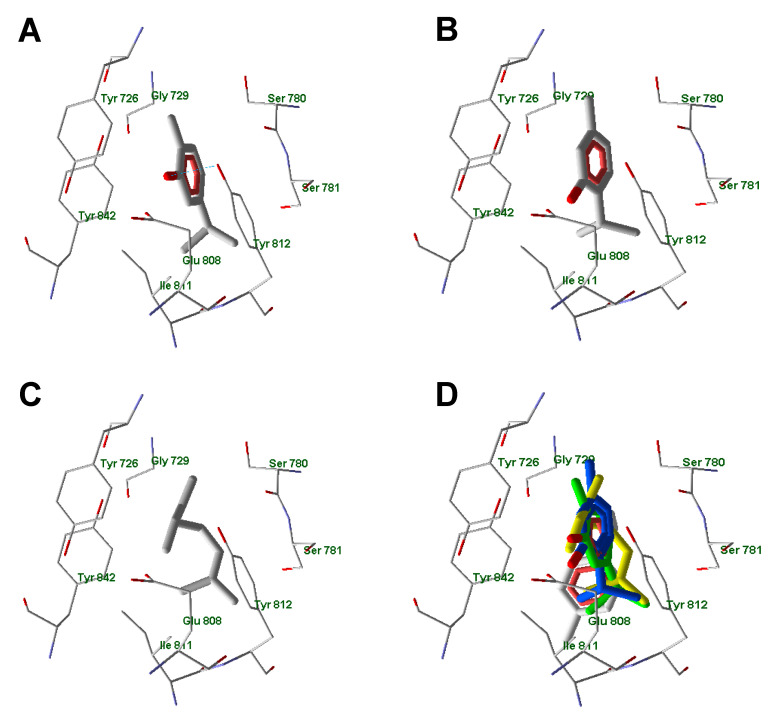
Docking poses of carvacrol (**A**), thymol (**B**), and β-myrcene (**C**). Superimposed docking poses of carvacrol (green skeleton), thymol (blue skeleton), β-myrcene (yellow), and inactive compound *p*-cymene (grey) (**D**). Residues within 6 Å of the center of the search space are shown.

We performed molecular docking studies on carvacrol, thymol, β-myrcene, and *p*-cymene using the human TRPA1 ion channel structure determined by electron cryo-microscopy (Protein Data Bank entry 3J9P [60]) as the target. The best docking pose for carvacrol (Figure 7) was nearly the same as that obtained by Alvarenga and co-authors [61] using the AutoDock program. We identified the formation of a H-bond between the phenolic hydroxyl and Tyr812 of the protein. However, this H-bond is quite weak, and its contribution to the docking score is −0.372. It should be noted that the isopropyl groups of carvacrol and thymol, as well as the (CH_3_)_2_C= fragment of β-myrcene, occupy the same area of space in the vicinity of the hydrophobic Ile811, Tyr812, and Tyr842 side chains. Hydroxyl groups of carvacrol and thymol are located close to each other in their superimposed docking poses and are directed to the OH and carboxyl groups of Tyr812 and Glu808, respectively. As for the inactive *p*-cymene, there was a noticeable difference in location of the molecule in the binding site when compared with the active compounds. There was a displacement of the *p*-cymene aromatic ring relative to the docking poses of the active aromatic compounds (carvacrol and thymol) and with respect to the location of the β-myrcene hydrocarbon chain.

## 3. Discussion

Essential oils isolated from *M. fistulosa* represent a rich source of bioactive monoterpenes with a broad range of therapeutic potential, as summarized in Table 1. The genus *Monarda* is represented by 20 native species, with *M. fistulosa* (wild bergamot) essential oils being used medicinally to treat cardiovascular defects, wounds, colds and flu, gastrointestinal disorders, insomnia, and infant convulsions [1]. Along with potent antimicrobial and antioxidant activity [16,62], Duke suggested that *M. fistulosa* also has anticarcinogenic properties [63]. In the present study, we defined the chemical profile of essential oils extracted from *M. fistulosa* flowers (MEO_Fl_) and leaves (MEO_Lv_). We further evaluated the agonist properties of these essential oils and their pure monoterpene constituents on TRPA1, TRPV1, and TRPV4 ion channels in heterologous cell expression systems.

A total of 45 compounds was isolated from both MEO_Fl_ and MEO_Lv_, with carvacrol, *p*-cymene, γ-terpinene, α-terpinene, thymol, and β-myrcene being the major constituents, along with 16 other compounds present in trace amounts (Table 2). As Mazza and Marshall indicated, the chemical composition of *M. fistulosa* essential oils is dependent on genotype and geographic origin [64]. Indeed, our results were comparable to the composition of *M. fistulosa* essential oils obtained from the Szczecin region in Poland, which had carvacrol, *p*-cymene, α-terpinene, and γ-terpinene as the major constituents [62] and were distinct from the high thymol content (60.8%) in *M. fistulosa* essential oils reported by Lawrence [65].

In recent years, extensive research has focused on the identification of bioactive ingredients and their cellular targets to explain the physiological benefits exerted by essential oils. TRP channels have emerged as potential targets for several essential oils that have health benefits, such as antinociception, anti-anxiolytic, anticonvulsant, anti-inflammatory, and immunomodulatory activities. To our knowledge, this is the first study investigating *M. fistulosa* essential oils and their effects on TRP channels. TRP channels are a family of 28 nonselective cation channels grouped into six subfamilies and exhibiting a high permeability to calcium. These channels are thermosensitive, nociceptive, and osmoregulatory, along with playing significant roles in vision, taste transduction, and hormone release. We focused on the TRPA1, TRPV1, and TRPV4 channels, based on previous reports of phytochemicals activating these channels with physiological significance. For example, cinnamaldehyde from cinnamon exerts beneficial effects on cardiovascular functions [48] and decreases blood sugar levels [49,50,51] through the activation of TRPA1 channels. Topical application of capsaicin, the active ingredient in chili peppers, has proven to be beneficial in treating arthritis [52] and diabetic peripheral neuropathy [53,54], possibly by the desensitization of TRPV1 channels. Likewise, eugenol, one of the active ingredients in basil and clove, exhibits anti-inflammatory properties by activating TRPV1 [66]. Both TRPV4 and TRPV1 have been suggested to contribute to paclitaxel-induced neuropathy [55]. Finally, our recent studies suggest that the activation of TRPV1 channels by *Ferula iliensis* and *Ferula akitschkensis* essential oils contributed to their neutrophil immunomodulatory activity [40].

MEO_Fl_ and MEO_Lv_ evoked a robust transient increase in [Ca^2+^]_i_ through TRPA1 channels. MEO_Lv_ exhibited a stronger activation profile than its flower counterpart, with 10 µg/mL of MEO_Lv_ eliciting significant transient increases in [Ca^2+^]_i_ compared to the negligible effect induced by the same concentration of MEO_Fl_. HC-030031, the specific reversible TRPA1 antagonist, completely blocked the MEO_Fl_- and MEO_Lv_-induced transient increases in [Ca^2+^]_i_, suggesting that future mechanistic studies involving TRPA1 modulation by essential oils in light of various diseases are warranted. The effects on TRPA1 channels were quite specific, as MEO_Fl_ and MEO_Lv_ induced negligible [Ca^2+^]_i_ changes in cells expressing functional hTRPV1 and hTRPV4 channels.

The major monoterpenes present in *M. fistulosa* essential oils, including carvacrol, β-myrcene, α-terpinene, γ-terpinene, and thymol, had no effect on either the hTRPV1 or hTRPV4 channels. Carvacrol has been implicated in a myriad of biological activities, including antioxidant, antimicrobial, and anti-inflammatory activities [67,68,69]. This monoterpene suppressed cyclooxygenase-2 expression [70] and was found to be an inhibitor of Drosophila TRPL and mammalian TRPM7 channels [71]. Consistent with our findings, Xu et al. [67] reported that carvacrol did not activate TRPV4 and induced only a small increase in [Ca^2+^]_i_ via TRPV1 channels expressed in tongue epithelial cells. Additionally, the same study showed that carvacrol robustly activated TRPA1 channels in free nerve endings of the tongue and nose [67]. Likewise, we observed that micromolar concentrations of pure carvacrol elicited a marked [Ca^2+^]_i_ increase in rTRPA1-expressing HEK293 cells (Figure 5A).

The other major constituent of *M. fistulosa* essential oils, thymol, is a phenolic monoterpene predominantly found in thyme/oregano oil. Thymol is an isomer and shares similar biological properties with that of carvacrol [68,72,73,74,75,76,77]. We found that thymol induced a transient increase in [Ca^2+^]_i_ through rTRPA1, which is similar to the enhancement of spontaneous excitatory transmission in substantia gelatinosa neurons reported by Xu et al. [78]. Additionally, several other studies have demonstrated the activation of TRPA1 channels by thymol [79,80,81].

Our study is also the first to show that β-myrcene activates TRPA1 channels. β-Myrcene found in lemongrass and basil has been traditionally used to treat diabetes, diarrhea, dysentery, and hypertension [82,83]. Transient increases in [Ca^2+^]_i_ elicited by β-myrcene were smaller in magnitude and demonstrated slower kinetics than those observed with carvacrol or thymol. A significant transient increase in [Ca^2+^]_i_ was elicited only by 125 µM of β-myrcene compared to 100 µM for carvacrol/thymol. Further studies need to be conducted to determine the exact EC_50_ value of β-myrcene at the TRPA1 channels, but this was beyond the scope of our present study. The only study investigating the “entourage effect” of terpenoids modulating the action of cannabinoids tested 10 µM of β-myrcene on hTRPA1-expressing HEK293 cells [84]. We found that a minimum dose of 125 µM was required to elicit significant increases in [Ca^2+^]_i_ through rTRPA1 channels in our heterologous cell expression system. Previous studies on the effects of α-terpinene and γ-terpinene on TRP channels are consistent with our results and showed that terpinenes did not induce significant responses through TRPA1, TRPV1, or TRPV4 channels [85].

The validation of our cell system provides us with confidence that these cells are reliable tools to study rTRPA1, hTRPV1, and hTRPV4 functional activation, and our stably transfected cells consistently responded to TRP agonists, AITC (TRPA1), capsaicin (TRPV1), and GSK-1019670A (TRPV4). However, β-myrcene failed to activate hTRPV1 in our study, contradicting a previous report [86]. There are numerous differences in assay conditions that may help explain the discrepant findings, including differences in expression levels of TRPV1, which may render other systems more sensitive to the effects of terpenoids, such as β-myrcene. Differences in the methods utilized to measure changes in [Ca^2+^]_i_, the temperature at which the experiments were performed, and the concentration of solvents might also contribute to the differences in the findings [87,88].

Carvacrol, thymol, and β-myrcene activated TRPA1 channels in the micromolar range, with selectivity for TRPA1 versus TRPV1 and TRPV4, implying that their pungent properties are mainly, if not exclusively, due to TRPA1 channel stimulation. Additionally, there have been a few reports implicating the involvement of other TRP channels as molecular targets for these monoterpenes. For example, Xu et al. suggested TRPV3 as a molecular target for skin sensitization by plant derivatives (carvacrol and thymol) isolated from oregano, thyme, savory, and clove [67]. Previous studies from our lab also demonstrated that thymol and carvacrol can activate TRPM8 [85]. Thymoquinone is also a significant constituent of *M. fistulosa L.* (Table 1). Interestingly, essential oils extracted from *Nigella sativa* (black cumin) seeds have therapeutic properties, including antitumor activity in multiple types of cancer [45,89], and Mihara et al. proposed a mechanism of interaction between TRPA1 and thymoquinone via Michael addition [90].

In conclusion, we report that essential oils isolated from the leaves and flowers of *M. fistulosa* are TRPA1 agonists. Moreover, we found that the *Monarda* essential oil constituents thymol, carvacrol, and β-myrcene were the primary mediators of TRPA1 channel activation. In support of these results, molecular modeling studies showed that these active molecules had similar poses within the binding site of human TRPA1. The interaction of these essential oils and constituents with TRPA1 channels provides a molecular basis to partially explain the therapeutic effects of *M. fistulosa* essential oils. We expect that future in vivo studies investigating the modulation/regulation of these TRP channels will enhance our understanding of the physiological effects of essential oils extracted from *M. fistulosa*.

## 4. Materials and Methods

### 4.1. Plant Material

*Monarda fistulosa* was collected in the month of August 2019 during the flowering and fruiting stages on the south side of Baldy Mountain, Gallatin Valley, MT, USA (45.7674° N, 110.9438° W) at an elevation of ~1750 m above sea level. Flowers and leaves were air-dried for 7–10 days at room temperature away from direct sunlight before hydrodistillation. Botanical identification of the plant material was performed by botanist Robyn Klein (Montana State University, Bozeman, MT, USA), who has extensive experience in the taxonomical identification of *Lamiaceae* species.

### 4.2. Chemical Reagents

Dimethyl sulfoxide (DMSO), α-terpinene, myrcene, (−)-limonene, (+)-limonene, and *p*-cymene were purchased from Acros Organics (Fair Lawn, NJ, USA). Carvacrol and thymol were purchased from Tokyo Chemical Industry Co. (Tokyo, Japan). Camphene, γ-terpinene, (−)-linalool, sabinene, and α-terpineol were from Sigma-Aldrich Chemical Co. (St. Louis, MO, USA). Terpinolene and α-pinene (mixture of isomers) were from Santa Cruz Biotech (Dallas, TX, USA), (1*S*)-(−)-β-pinene and terpinen-4-ol were from Alfa-Aesar (Ward Hill, MA, USA), and *n*-hexane was purchased from Merck (Darmstadt, Germany). TRPA1 antagonist HC-030031 was from Tocris Bioscience (Ellisville, MS, USA). Stock concentrations of monoterpenes were prepared in DMSO. Hanks’ balanced salts (HBSS) were purchased from Sigma-Aldrich (H1387; St. Louis, MO, USA). The calcium indicator dye Fura-2 acetoxy methylester (Fura 2-AM) was purchased from AAT-Bioquest (Sunnyvale, CA, USA). Working concentrations of treatments were prepared in HBSS.

### 4.3. Essential Oil Extraction

Essential oils were obtained by hydrodistillation of dried plant material using a Clevenger-type apparatus, as previously described [59]. We used conditions accepted by the European Pharmacopoeia (European Directorate for the Quality of Medicines, Council of Europe, Strasbourg, France, 2014) to avoid artifacts. The yield of the essential oil was calculated based on the amount of air-dried plant material used. Stock solutions of the essential oils were prepared in DMSO (10 mg/mL) for biological evaluation.

### 4.4. Gas Chromatography-Mass Spectrometry (GC-MS) Analysis

Essential oil samples were dehydrated using anhydrous Na_2_SO_4_ and then suspended in *n*-hexane (10% w/v) for GC-MS analysis, which was performed with an Agilent 5975 GC-MSD system (Agilent Technologies, Santa Clara, CA, USA), as reported previously [91]. An Agilent Innowax FSC column (60 m × 0.25 mm, 0.25-μm film thickness) was used with He as the carrier gas (0.8 mL/min). The GC oven temperature was kept at 60 °C for 10 min, increased to 220 °C at a rate of 4 °C /min, kept constant at 220 °C for 10 min, and then increased to 240 °C at a rate of 1 °C /min. The split ratio was adjusted to 40:1, and the injector temperature was 250 °C. MS spectra were monitored at 70 eV with a mass range of 35 to 450 *m*/*z*.

GC analysis was performed using an Agilent 6890N GC system. To obtain the same elution order as with GC-MS, the line was split for FID and MS detectors, and a single injection was performed using the same column and appropriate operational conditions. Flame ionization detector (FID) temperature was 300 °C. The essential oil components were identified by co-injection with standards (whenever possible), which were purchased from commercial sources or isolated from natural sources. In addition, compound identities were confirmed by comparison of their mass spectra with those in the Wiley GC-MS Library (Wiley, NY, USA), MassFinder software 4.0 (Dr. Hochmuth Scientific Consulting, Hamburg, Germany), Adams Library, and National Institutes of Standards and Technology (NIST) Library. Confirmation was also achieved using the in-house “Başer Library of Essential Oil Constituents” database obtained from chromatographic runs of pure compounds performed with the same equipment and conditions. A C_8_–C_40_ *n*-alkane standard solution (Fluka, Buchs, Switzerland) was used to spike the samples for the determination of the relative retention indices (RRI). Relative percentage amounts of the separated compounds were calculated from FID chromatograms.

### 4.5. Heterologous Expression of TRP Channels in HEK 293 Cells

HEK 293 cells transfected with rat TRPA1 (HEK rTRPA1) or human TRPV1 (HEK hTRPV1) were a generous gift from Dr. Luciano De Petrocellis (ICB-CNR, Naples, Italy). Cells were cultured in minimum essential medium (MEM) (Gibco, Madison, WI, USA) supplemented with 10% fetal bovine serum (FBS) (Hyclone Labs, Logan, UT, USA), 1% Pen-Strep (Gibco), 1% nonessential amino acids (Gibco), and 2-mM Glutamax (Gibco) and maintained under 5% CO_2_ at 37 °C. Following the second passage, the growth medium was supplemented with 0.6-mg/mL geneticin/G418 (VWR, Randor, PA, USA) to induce the stable expression of the TRPA1 and TRPV1 ion channels.

HEK 293 cells transfected with human TRPV4 (HEK hTRPV4) were a generous gift from Dr. Sara Baratchi (Stanford University, CA, USA). Cell culture medium was comprised of Dulbecco’s modified Eagle’s medium (DMEM) (4.5-g/L glucose) supplemented with 10% FBS, 1-mg/mL blasticidine (InvivoGen, San Diego, CA, USA), and 50-mg/mL hygromycin (InvivoGen) for the selection of stably expressing cells and maintained under 5% CO_2_ at 37 °C. Following the first passage, cells were induced overnight with 0.1-µg/mL tetracycline for the expression of TRPV4 channels. Parental HEK 293 cells were purchased from the American Type Culture Collection (ATCC) and cultured according to the manufacturer’s instructions and were used as experimental controls.

### 4.6. “Real-Time” [Ca^2+^]_i_ Measurements

For real-time [Ca^2+^]_i_ measurements, all cell types were grown as monolayers until 70–80% confluency. Cells were incubated at 37 °C for 30 min with 5-µM Fura 2-AM in complete medium, followed by two gentle washes. De-esterification was allowed for a further 30 min, and then, the monolayers were mounted on the stage of an Olympus IX-81 inverted fluorescence microscope (Olympus America, Center Valley, PA, USA). Cells were superfused continuously with HBSS (in mM: 118 NaCl, 4.8 KCl, 1.23 CaCl_2_, 0.6 KH_2_PO_4_, 4.6 NaHCO_3_, 0.6 NaH_2_PO_4_, and 5.5 glucose, pH 7.4) at a flow rate of 2 mL/min. Cells were exposed to compounds under investigation by switching from the control medium (HBSS with Ca^2+^) to the compound-containing medium (working concentrations of treatment prepared in HBSS with Ca^2+^) for 30s, unless noted otherwise. Control buffer and treatments were always kept in separate chambers to prevent any potential interaction between the compounds. Relative [Ca^2+^]_i_ was allowed to return to baseline after a response before exposing the cells to any successive treatment. Cells were pretreated with the TRPA1 antagonist HC-030031 (20 µM) for 20 min to block TRPA1 activation with essential oils. ATP (25 µM) was routinely utilized to confirm receptor-effector coupling in control parental HEK 293 cells. The [Ca^2+^]_i_ measurements were recorded in individual cells using a fluorescence imaging system (Easy Ratio Pro software, Horiba Scientific, North Edison, NJ, USA) equipped with a multi-wavelength DeltaRAM monochromator (Photon Technology International, Inc., Edison, NJ, USA) and a QuantEM 512SC electron multiplying charge-coupled device camera (Teledyne Photometrics, Tucson, AZ, USA), as previously described [40]. Images and real-time [Ca^2+^]_i_ data were acquired using an alternating excitation wavelength protocol (340 nm and 380 nm, 20 Hz) and emission wavelength of 510 nm. Background fluorescence was automatically corrected for the experiments using Easy Ratio Pro software. The signals obtained at 340 and 380 nm were calculated as the 340/380 ratio and used as an index to measure relative changes in [Ca^2+^]_i_ from the baseline [92] and expressed as mean ± SEM.

### 4.7. Molecular Docking

The structure of TRPA1 determined experimentally by electron cryo-microscopy [60] was downloaded from the Protein Data Bank (PDB code 3J9P) and imported into the Molegro Virtual Docker (MVD) program. The search space for docking was defined as a sphere of 8 Å in radius positioned at the geometric center of six residues in chain A (Tyr726, Ser780, Ser781, Ile811, Tyr812, and Tyr842) surrounding the reported binding site [61] for the docking of carvacrol. The spherical search space contained 26 residues (Ser725, Tyr726, Gly729, Leu730, Met733, Val777, Ser780, Ser781, Ile782, Phe783, Gly784, Leu807, Glu808, Ile810, Ile811, Tyr812, Thr813, Thr814, Gly815, Ala838, Phe841, Tyr842, Trp843, Asn845, Phe846, and Tyr849). Among these, Leu730 and Glu808 were not fully resolved in the experimental 3J9P structure downloaded from the PDB. These two residues were repaired and locally optimized using the “Protein preparation” utility incorporated in MVD.

Three-dimensional conformers of carvacrol, thymol, β-myrcene, and *p*-cymene were downloaded from the PubChem database (compound ID numbers 10364, 6989, 31253, and 7463, respectively) and imported into MVD. Docking was performed with a rigid receptor and flexible ligands. For each compound, 300 docking runs were performed. For the docking poses obtained, additional geometry optimizations were carried out using the “Energy Minimization” and “Optimize HBonds” options of the MVD program.

### 4.8. Statistical Analyses

The data are expressed as mean ± SEM. Statistical comparisons were made with an unpaired *t*-test, one-way ANOVA, and two-way ANOVA, as appropriate. For all statistical analyses, GraphPad Prism 8.0 software was used (GraphPad, San Diego, CA, USA). *p*-values are as stated in the figure legends.

## Figures and Tables

**Table 1:** Biological activity of essential oils of *Monarda* species and their major constituents (literature survey).

Monarda spp.	Biological Activity	Major Constituents	Ref.
*M. didyma* L.	Nematotoxic	Carvacrol, γ-terpinene, *o*-cymene, and thymol	[11]
	Antifungal	Thymol, carvacrol, α-terpinene, *p*-cymene, γ-terpinene	[12,13]
	Phytotoxic	Essential oil	[14]
	Antimicrobial	Thymol	[10]
	-	Thymol, thymol methyl ether	[8]
*M. fistulosa* L.	Nematotoxic	Carvacrol, γ-terpinene, *o*-cymene, and thymol	[11]
	Antiseborrheic	Essential oil	[15]
	Repellent	Carvacrol, thymol, eugenol, carvacrol methyl ether	[16]
	-	Thymol, carvacrol, thymoquinone	[17,18]
	Antifungal	Geraniol, geranial, geranyl formate, *p*-cymene	[13]
	Antioxidant	Essential oil, thymol, carvacrol	[19]
	Antimicrobial	Thymol	[10]
*M. fistulosa, M. didyma*	-	Geraniol, linalool, thymol, carvacrol, 1,8-cineole	[20]
*M. bradburiana* Beck	Repellent	Carvacrol, carvacrol methyl ether, thymol, eugenol	[16]
*M*. *punctata L.*	Antilipase	Carvacrol	[7]
	Antibacterial	Thymol, *p*-cymene, limonene, carvacrol	[24]
	Fungicidal	Thymol	[22]
*M. citriodora* Cerv. ex Lag.	Antimicrobial	Essential oil, hydrolate	[23]
	Antibacterial	Thymol, 1,8-cineole, α-phellandrene, *p-*cymene	[24]
	Antifungal	β-Caryophyllene, citral, limonene, *cis*-verbenol	[25]
	Anticancer (*in vitro*)	Essential oil, thymol	[26]
*M. citriodora var. citriodora* Cerv. ex Lag.	Antioxidant, antimicrobial	Essential oil	[27]
*M. menthifolia* Graham	-	*p*-Cymene, carvacrol, hydrothymoquinone, geraniol, linalool	[29]
*M. pectinata* Nutt.	-	Carvacrol	[29]
*Monarda* spp.	Antiplasmodial	Essential oil, thymoquinone	[28]

**Table 2:** Chemical composition of *M. fistulosa* essential oils isolated from flowers (MEO_Fl_) and leaves (MEO_Lv_).

#	RRI	Compound	MEO_Fl_	MEO_Lv_	#	RRI	Compound	MEO_Fl_	MEO_Lv_
1	1032	α-Pinene	0.7	0.4	23	1497	α-Copaene	T	t
2	1035	α-Thujene	2.4	1.3	24	1535	β-Bourbonene	T	0.1
3	1076	Camphene	0.1	0.1	25	1553	Linalool	0.1	0.1
4	1118	β-Pinene	0.2	0.1	26	1556	*cis*-Sabinene hydrate	0.2	0.2
5	1132	Sabinene	0.3	0.1	27	1571	*trans-p*-Menth-2-en-1-ol	0.1	t
6	1159	δ-3-Carene	0.2	0.1	28	1590	Bornyl acetate	T	
7	1167	3-Heptanone	t		29	1611	Terpinen-4-ol	1.1	
8	1174	β-Myrcene	1.4	0.8	30	1614	Methyl carvacrol	1.1	1.4
9	1176	α-Phellandrene	0.3	0.2	31	1638	*cis-p*-Menth-2-en-1-ol	T	
10	1183	*p*-Mentha-1,7(8)-diene	t		32	1689	*trans*-Piperitol	0.2	
11	1188	α-Terpinene	3.8	1.7	33	1704	γ-Muurolene		t
12	1203	Limonene	0.9	0.5	34	1706	α-Terpineol	0.2	0.1
13	1218	β-Phellandrene	0.3	0.2	35	1719	Borneol	0.2	0.1
14	1255	γ-Terpinene	6.8	2.5	36	1726	Germacrene D	0.3	0.2
15	1266	3-Octanone	t		37	1773	δ-Cadinene		t
16	1280	*p*-Cymene	25.6	13.1	38	1802	Cumin aldehyde		t
17	1290	Terpinolene	0.1		39	1864	*p*-Cymen-8-ol		0.1
18	1393	3-Octanol	0.1		40	1889	Ascaridole		t
19	1400	Nonanal	t		41	1940	4-Isopropyl salicylaldehyde		t
20	1408	1,3,8*-p-*Menthatriene	t		42	1969	*cis*-Jasmone		0.1
21	1452	1-Octen-3-ol	1.5	0.9	43	2113	Cumin alcohol		t
22	1474	*trans*-Sabinene hydrate	1	0.8	44	2198	Thymol	3.1	3.3
					45	2239	Carvacrol	45.7	71.5

The data are presented as relative % by weight for each component that was isolated from *M. fistulosa* flowers and leaves. RRI, relative retention index calculated on the basis of retention of *n*-alkanes; %, calculated from flame ionization detector data. Trace amounts (t) were present at <0.1%. # means compound number.

**Table 3:** Summary of the chemical compositions of MEO_Fl_ and MEO_Lv_.

Total	MEO_Fl_	MEO_Lv_
	%	
	99.8	100
Monoterpene hydrocarbons	43.1	21.1
Oxygenated monoterpenes	54.8	77.7
Sesquiterpene hydrocarbons	0.3	0.3
Miscellaneous compounds	1.6	0.9

**Table 4:** Relative content of constituent monoterpenes in MEO_Fl_ and MEO_Lv_ and their effects on [Ca^2+^]_i_ flux in HEK rTRPA1-transfected cells.

Compound	Composition		HEK Cells	
	MEO_Fl_	MEO_Lv_	rTRPA1	NT
	%		[Ca^2+^]_i_ Change	
α-Pinene	0.7	0.4	0.054 ± 0.014	0.013 ± 0.002
Camphene	0.1	0.1	0.014 ± 0.004	0.009 ± 0.001
β-Pinene	0.2	0.1	0.012 ± 0.002	0.011 ± 0.001
Sabinene	0.3	0.1	0.029 ± 0.005	0.034 ± 0.004
β-Myrcene	1.4	0.8	0.111 ± 0.003	0.005 ± 0.001
α-Terpinene	3.8	1.7	0.007 ± 0.001	0.006 ± 0.001
(-) Limonene	0.9	0.5	0.008 ± 0.002	0.007 ± 0.001
(+) Limonene	-	-	0.011 ± 0.002	0.009 ± 0.002
γ-Terpinene	6.8	2.5	0.034 ± 0.005	0.014 ± 0.001
*p*-Cymene	25.6	13.1	0.071 ± 0.011	0.027 ± 0.001
Terpinolene	0.1	-	0.034 ± 0.007	0.022 ± 0.003
Linalool	0.1	0.1	0.037 ± 0.007	0.002 ± 0.0003
Terpinen-4-ol	1.1	-	0.046 ± 0.007	0.013 ± 0.002
α-Terpineol	0.2	0.1	0.010 ± 0.002	0.004 ± 0.001
Thymol	3.1	3.3	0.849 ± 0.013	0.026 ± 0.002
Carvacrol	45.7	71.5	0.340 ± 0.012	0.061 ± 0.018

Values of [Ca^2+^]_i_ changes are presented as the mean ± S.E.M. of 3 independent experiments, as described in Materials and Methods. The cells were treated with 100 µM of monoterpene, and [Ca^2+^]_i_ was monitored, as described. NT, nontransfected HEK cells.
